# CoRAL: predicting non-coding RNAs from small RNA-sequencing data

**DOI:** 10.1093/nar/gkt426

**Published:** 2013-05-21

**Authors:** Yuk Yee Leung, Paul Ryvkin, Lyle H. Ungar, Brian D. Gregory, Li-San Wang

**Affiliations:** ^1^Department of Pathology and Laboratory Medicine, Perelman School of Medicine, University of Pennsylvania, Philadelphia, PA 19104, USA, ^2^Penn Center for Bioinformatics, Perelman School of Medicine, University of Pennsylvania, Philadelphia, PA 19104, USA, ^3^Genomics and Computational Biology Graduate Group, Perelman School of Medicine, University of Pennsylvania, Philadelphia, PA 19104, USA, ^4^Department of Computer and Information Science, University of Pennsylvania, Philadelphia, PA 19104, USA, ^5^Penn Genome Frontiers Institute, Perelman School of Medicine, University of Pennsylvania, Philadelphia, PA 19104, USA, ^6^Department of Biology, University of Pennsylvania, Philadelphia, PA 19104, USA and ^7^Institute on Aging, Perelman School of Medicine, University of Pennsylvania, Philadelphia, PA 19104, USA

## Abstract

The surprising observation that virtually the entire human genome is transcribed means we know little about the function of many emerging classes of RNAs, except their astounding diversities. Traditional RNA function prediction methods rely on sequence or alignment information, which are limited in their abilities to classify the various collections of non-coding RNAs (ncRNAs). To address this, we developed Classification of RNAs by Analysis of Length (CoRAL), a machine learning-based approach for classification of RNA molecules. CoRAL uses biologically interpretable features including fragment length and cleavage specificity to distinguish between different ncRNA populations. We evaluated CoRAL using genome-wide small RNA sequencing data sets from four human tissue types and were able to classify six different types of RNAs with ∼80% cross-validation accuracy. Analysis by CoRAL revealed that microRNAs, small nucleolar and transposon-derived RNAs are highly discernible and consistent across all human tissue types assessed, whereas long intergenic ncRNAs, small cytoplasmic RNAs and small nuclear RNAs show less consistent patterns. The ability to reliably annotate loci across tissue types demonstrates the potential of CoRAL to characterize ncRNAs using small RNA sequencing data in less well-characterized organisms.

## INTRODUCTION

One of the most significant biological discoveries of the past decade includes the discovery of new types of RNAs and their specific functions in eukaryotic cells (1,2). For instance, non-coding RNAs (ncRNAs) are transcripts that are not translated into proteins but serve other important biological functions. ncRNAs have highly diverse functions including protein translation [transfer RNAs (tRNAs) and ribosomal RNAs], regulation of gene expression [microRNAs (miRNAs) and long intergenic non-coding RNAs (lincRNAs)] (3,4), pre-mRNA splicing [small nuclear RNAs (snRNAs)] (5), RNA modification [small nucleolar RNAs (snoRNAs)] (6) and the list is still expanding. Advances in high-throughput sequencing technologies have led to the unexpected discovery that up to 93% of the human genome is transcribed in some tissues (7). Thus, it is not surprising that the ncRNA database (8) includes 135 different ncRNA classes. Unfortunately, the classification of most RNAs in this database is more representative of the historical process by which the ncRNAs were discovered, such as sedimentation coefficient (e.g. 4.5S RNA) or cellular location (e.g. snoRNA), than of their true cellular functions. This gap highlights the fact that most transcribed regions are still of unknown molecular function and biological significance.

Given that little is known about most ncRNAs, a potential approach is to gather an enormous amount of experimental data efficiently and systematically using RNA sequencing (RNA-seq) and to analyse these data using sophisticated computational approaches. Unlike microarrays, RNA-seq does not rely on target probe hybridization, and thus one does not need to know in advance which regions are being transcribed. These properties make RNA-seq a promising tool to study ncRNA biology. Additionally, RNA-seq is highly versatile in that it can be modified to study specific properties, e.g. small RNA-seq (smRNA-seq) (9) where gel-based size selection is used to enrich RNAs with particular sequence lengths.

While traditional methods predict RNA function using primary sequence or alignment information, new approaches using RNA-seq data have been proposed. For example, the miRDeep2 algorithm (10) searches for genomic regions that fold into hairpin structures and are enriched for sequenced reads next to the hairpin loop region (the expected location of mature miRNAs) to identify potential miRNA loci. Additionally, Langenberger *et al.* (11) pioneered the use of smRNA-seq features such as abundance and block length distribution to classify ncRNAs. Their method DARIO (12) uses random forest (RF) classifiers to differentiate between miRNA, snoRNA and tRNA, loci with reasonable performance. However, features generated from DARIO are not normalized by transcript-wide abundance, and as a result, the most informative feature for miRNA identification is their overall abundance. This does not generalize to other ncRNAs and is simply a result of the fact that miRNAs are highly abundant in human smRNA-seq data sets.

Erhard and Zimmer (13) used similarities between RNA transcripts to classify ncRNAs. Their similarity measure was created based on the relative positions and lengths obtained from sequencing experiments. However, relative positions of reads require good knowledge on the start- and end-points of transcripts within a genome sequence, which is a challenge for newly discovered classes of ncRNA. Evaluation of their method on two classes of RNA (miRNAs and tRNAs) yielded performance with recall values of 98% and precision of 60% for miRNAs and ∼80% for tRNAs, which leaves room for improvement.

**Figure 1.**
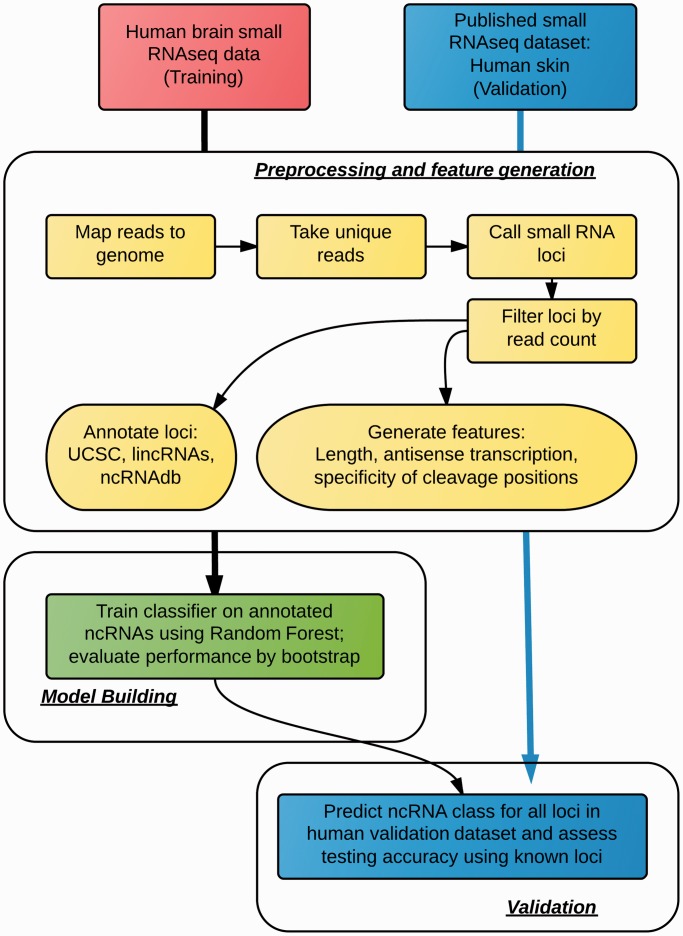
The analysis workflow for differentiating between six different classes of ncRNAs in smRNA-seq data sets.

To address the limitations of these previous RNA function classifiers, we have developed a framework for classifying RNA transcripts by functional categories using smRNA-seq data (Figure 1), which can then be applied to identify unannotated RNAs with similar functions in other organisms in the future. To do this, we first designed algorithms to generate several types of features from smRNA-seq data based on read length distribution, strand specificity and the secondary structure of the transcript for transcribed genomic regions. We then applied a multi-class classification algorithm with feature selection and cross-validation schemes included to train classifiers among a collection of known RNA functional classes including lincRNAs, miRNAs, small cytoplasmic RNA (scRNAs), C/D box snoRNAs, snRNAs and transposon-derived RNAs. For each RNA class, we identified the most informative features that might be associated with the molecular mechanisms and metabolic processes of the functional classes. Trained models, informative features and annotation results have been validated using (i) external datasets, (ii) SAVoR, a visualization tool for RNA structures (14), and (iii) curation of the primary literature.

## MATERIALS AND METHODS

### Processing of small RNA-seq data

The smRNA-seq data used for our analysis came from four sources: human brain data generated as part of this study (GSE43335), a previously published data set from human skin (GSE31037) (15) and published data sets from human liver (SRR040571) and muscle (SRR040572) (16) (Table 1). The human brain data were obtained by sequencing small RNAs (smRNAs) extracted from the dorsolateral prefrontal cortex of four deceased human patients with no apparent pathology. All reads were trimmed to remove the Illumina 3′ adapter sequence using cutadapt (17), and only those reads containing the adapter were taken as true smRNA reads. Reads were mapped to the reference genome GRCh37/hg19 using Bowtie (18) and those mapping to multiple loci were discarded. To merge reads into transcribed loci, we used the RSEQTools’ (19) bgrSegmenter tools.

**Table 1.** Number of reads in the four smRNA-seq data sets at various stages of processing, ordered from left to right

Tissues	Raw reads	3’ adapter trimmed reads	Uniquely mapped reads	smRNA loci, ≥ 1 read	smRNA loci, ≥ 15 reads
Brain	104 120 855	51 929 478 (50%)	15 401 850 (30%)	6246	4525 (72%)
Skin	307 025 425	188 417 173 (61%)	85 443 864 (28%)	11 423	8638 (76%)
Liver	3 374 986	1 477 497 (44%)	1 152 829 (78%)	269	216 (80%)
Muscle	3 793 410	3 417 173 (90%)	368 271 (11%)	218	178 (82%)

### Labelling training data

Functional categories were assigned to loci by overlapping their coordinates with RNA annotations from the UCSC Genome Browser (20). Although there are many different types of ncRNA described, we focused on a subset of functional classes where sufficient numbers of confirmed loci were available to train predictive models.

**Figure 2.**
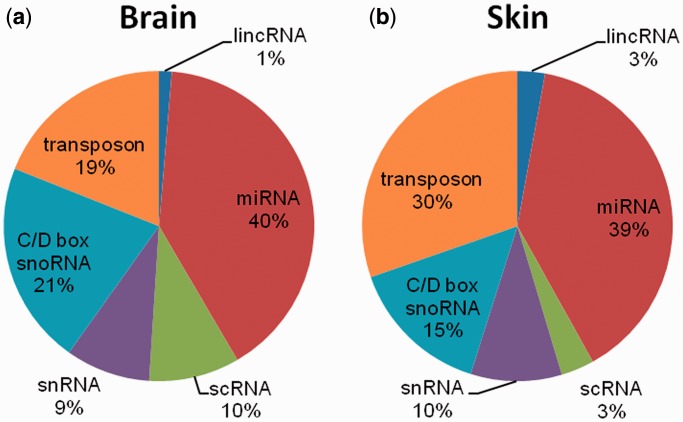
Percentage of small ncRNA loci identified by smRNA-seq for two human tissue types: (**a**) brain and (**b**) skin.

For quality control purposes, loci covered by fewer than 15 reads were discarded. This value was chosen as a compromise between selecting high-quality sufficiently transcribed regions and identifying significant levels of loci for each class (Supplementary Figure S1). Based on these criteria, the following six RNA classes were selected: lincRNAs, miRNAs, scRNAs, C/D box snoRNAs, snRNAs and transposon-derived RNAs (Figure 2). We excluded ribosomal RNAs and tRNAs because they are easily identifiable by sequence homology alone.

### Feature generation

We noted that features used for classification purposes should be flexible, comprehensive, efficient and scalable. Therefore, we developed features that would most likely be used to reflect the underlying biological properties of small ncRNAs. For example, miRNAs are consistently processed into their mature form of 22 nucleotide (nt) fragments as a consequence of Dicer’s activity on the stem-loop structure of pre-miRNAs (21). It is reasonable to assume, then, that the lengths of smRNAs are consistent with some aspects of their biogenesis, which should also be consistent within classes sharing the same molecular function. Thus, for a transcribed locus *i* that starts at genomic position *a* and ends at position *b*, we define the length features as:

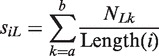

for read lengths 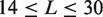, where *N_Lk_* is the number of reads of length *L* mapping to base *k* and Length (*i*) is the length of locus *i*. The values of these 17 features are then transformed into log-odds ratios via the following normalization procedure:

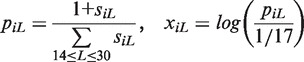



In addition to the read lengths, we introduced a feature based on the abundance of antisense transcription. The numerical value of this feature reflects the number of reads mapped to the antisense strand of the transcribed locus. This feature is generated based on the assumption that the presence of antisense transcription at a locus is relevant to the biogenesis of smRNAs from this region. Another important feature that is likely to be specific to smRNA biogenesis is the specificity of cleavage positions. We encode this as two features: 5′ and 3′ positional entropy. The entropy is computed based on the distributions of the 5′ and 3′ end positions of all smRNA reads mapped to a given locus, respectively. This entropy feature is designed to capture the specificity (or degeneracy) of RNA cleaving enzymes specific to the production of different types of smRNAs. For example, the processing of mature miRNAs from pre-miRNAs tends to produce fragments with a more stable 5′ cleavage position (low entropy) and more variable 3′ end (higher entropy). We also generate features corresponding to the base composition of the reads, weighted by their expression: these are the four nucleotide frequencies transformed into a log-odds ratio relative to equal base frequencies. Additionally, we compute the predicted minimum free energy (MFE) of the genomic region surrounding the transcribed locus (40 bp on either side) using RNAfold with default parameters (22).

### Feature selection and classification framework

To identify features that are most representative of the six ncRNA classes, we used the R package varSelRF (version 0.7-3) (23), which selects a small optimal set of non-redundant features for each class. When computing the feature importance, we used varSelRF with parameters (mtryFactor = 4, vars.drop.fac = 0.35, ntree =1000). For the number of variables mtryFactor setting, we tried various values and saw no difference in performance; therefore, we used a value corresponding to the square root of the number of features as recommended in the literature (24). Similarly, the number of trees did not greatly affect accuracy but had a large impact on running time. The selected variable drop factor yielded classifiers with the highest training accuracy. RF was used as a classifier to distinguish between multiple RNA classes. The feature selection portion uses both backwards variable elimination and selection based on the variable importance index outputted by the RF model. When training the models, 100 RF models comprising 1000 trees were built to determine the stability of results.

### Evaluation of performance

Typically, the performance of a binary-class classifier is evaluated by comparing values of the confusion matrix, including rates of true positives (TP), true negatives (TN), false positives (FP) and false negatives (FN). Other commonly used measures for binary classification are accuracy, recall/sensitivity and positive predictive value (PPV). Measures for multi-class classification are generalized from measures used in binary classification. ACC_k_ is the overall accuracy, which is the proportion of predictions that are correct: ACC_k_ = (TP_k_ + TN_k_)/(TP_k_ + TN_k_ + FP_k_ + FN_k_). For every class C_k_, the class-specific evaluation measures are defined by recall (REC_k_) and PPV_k_, derived from counts of C_k_ from the confusion matrix. REC_k_ is defined as the proportion of positive labelled samples that are predicted as positive: REC_k_ = TP_k_/(TP_k_ + FN_k_), whereas PPV_k_ is defined as the proportion of positive samples that are correctly identified: PPV_k_ = TP_k_/(TP_k_ + FP_k_).

## RESULTS AND DISCUSSION

### Visualization of the length features

**Figure 3.**
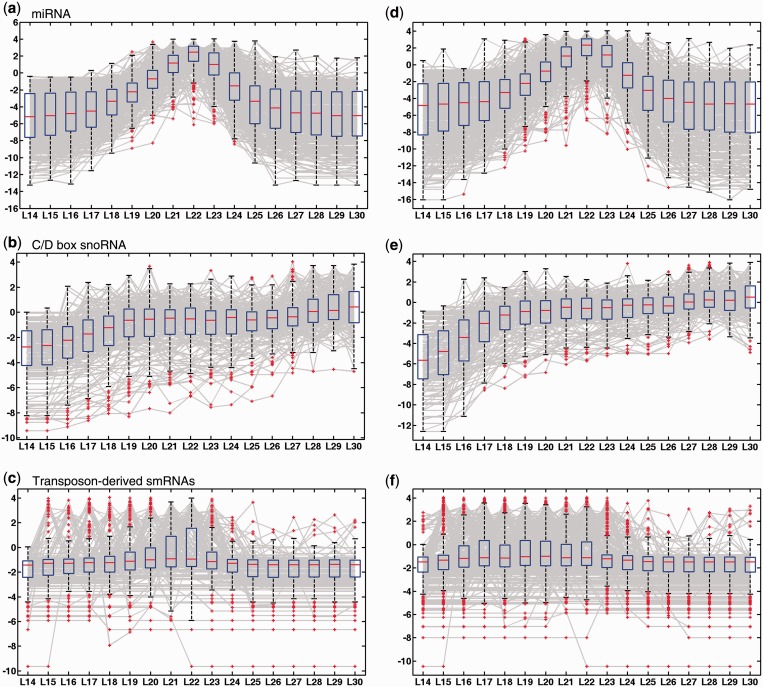
Feature spectrum plots for three of the ncRNA classes (as specified in the figure), in the (**a–c**) brain data and (**d–f**) the skin data. Each box corresponds to one length feature, and each grey line represents one locus. The red dots are outside of the 99th percentile of each distribution.

**Figure 4.**
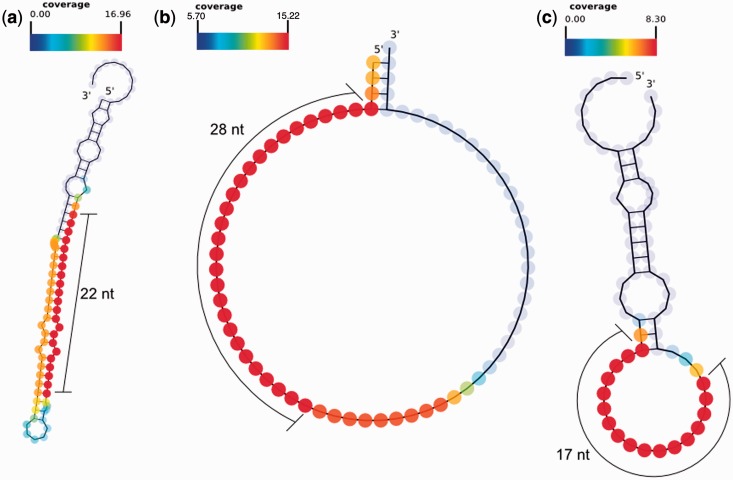
smRNA-seq reads plotted on the predicted RNA secondary structures using SAVoR (14) for (**a**) an miRNA, (**b**) a C/D box snoRNA and (**c**) a transposon-derived RNA. The miRNA and C/D box snoRNA structures are as reported by RFAM, and the transposon-derived RNA structure is as predicted by RNAfold.

We hypothesized that the lengths of some small ncRNAs are specific to particular classes of precursor ncRNAs. Therefore, we tested the distribution of the read length feature for three of the ncRNA classes in the human brain and skin data sets (Figure 3, Supplementary Figure S2). miRNAs demonstrated a strong peak at 22 nt in length (Figures 3a and d and 4a), which is consistent with what is known about the length of mature miRNAs in animals. Products coming from C/D box snoRNAs tend to be depleted of shorter RNAs and enriched for longer RNAs (Figures 3b and e and 4b). Transposon-derived smRNAs appear to show slightly different distributions depending on the tissue type. For example, they show a weak broad peak ∼19–23 nt in the brain data and a flatter, weaker bias towards 16–22 nt in the skin data (Figures 3c and f and 4c).

In addition, we examined the correlations between the features in the brain data set (Supplementary Figure S3). Unsurprisingly, features corresponding to adjacent lengths correlate strongly. Interestingly, there appear to be four clusters of lengths: 14–18, 19–20, 21–23 and 24–30 nt. These results suggest that specific classes of smRNAs tend to have coherent lengths. We also found that positional entropy at both ends of human brain smRNAs strongly correlate. This suggests that smRNAs with high 5′ cleavage specificity also tend to have high 3′ cleavage specificity.

### Discriminative power of features

**Figure 5.**
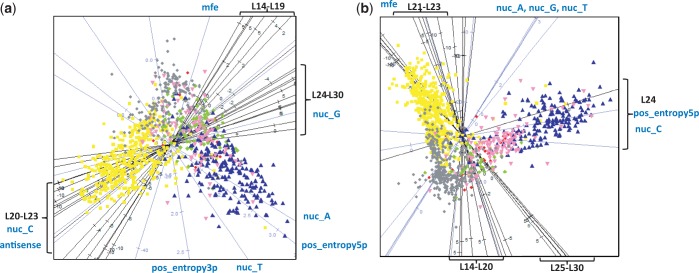
MDS based projections of the data for (**a**) brain and (**b**) skin. The three most discriminative classes are miRNA (yellow), C/D box snoRNA (blue) and transposon-derived RNAs (grey).

Owing to the varying number of loci within each ncRNA class, it was challenging to visualize all loci in a data set. To determine how well the length features were able to separate the loci, we built RF trees by classifying one ncRNA class versus all other classes. We then applied multidimensional scaling (MDS) to the proximity matrix obtained from the RF trees. miRNA, C/D box snoRNAs and transposon-derived RNAs were the most visually distinguishable classes of smRNAs using our features (Figure 5), and this pattern was found to be consistent between the two (brain and skin) data sets.

### Comparison with existing classification approaches—DARIO and miRDeep

We compared our method with a published method (DARIO), which was designed for classifying smRNAs by their precursor ncRNA loci. As DARIO only uses three classes of ncRNAs (miRNAs, C/D box snoRNAs and tRNAs) for building its classification model, we ran Classification of RNAs by Analysis of Length (CoRAL) while limiting the data to those three classes only (Table 2).

**Table 2.** Comparison of the performance of classification models built on three classes of ncRNAs in the brain data

	DARIO	CoRAL
miRNA		
REC (%)	90	94
PPV (%)	92	95
C/D box snoRNA		
REC (%)	N/A	88
PPV (%)	N/A	91
tRNA		
REC (%)	84	90
PPV (%)	81	87
Overall accuracy (%)	87	91

CoRAL gives the best results for all three classes, with an improvement of ∼3–4% for miRNAs and tRNAs. DARIO reported none of the loci as being annotated as snoRNAs, and so that class was unable to be compared, but demonstrates that CoRAL is able to identify these RNAs that cannot be distinguished by DARIO. When restricting the comparison with miRNAs and tRNAs, CoRAL’s predictive performance is 91%, which is a 4% improvement over the same analysis performed by DARIO.

Additionally, we compared our results with those produced by miRDeep2 on the brain data (ran with default parameters). miRDeep2 had a recall of 81% and PPV of 98%, whereas CoRAL had a recall of 88% and PPV of 91% for miRNAs while also predicting five other RNA classes. Thus, CoRAL has increased functional classification capabilities as well as improved overall performance compared with the currently available classifier options.

### Building a classification model using six classes of ncRNAs

There are currently >135 classes of ncRNAs in the NONCODE database. Here, using the two high-depth data sets, human brain and skin, we focused on a subset of functional classes where sufficient numbers of confirmed loci were available for us to build our predictive models. A total of six classes were included: lincRNAs, miRNAs, scRNAs, C/D box snoRNAs, snRNAs and transposon-derived smRNAs. Performance measures were averaged over 100 different seeds of RF classifiers (Table 3).

**Table 3.** Comparison of training (cross-validated) performance of RF models using the six ncRNAs studied in human brain and skin data

	Brain		Skin	
	CoRAL	Baseline	CoRAL	Baseline
lincRNA			
Count	13		34	
Recall (%)	16	0	1	1
PPV (%)	62	0	38	2
miRNA			
Count	397		465	
Recall (%)	91	78	89	71
PPV (%)	88	43	86	42
scRNA			
Count	93		41	
Recall (%)	78	1	29	0
PPV (%)	81	7	49	0
C/D box snoRNA			
Count	209		176	
Recall (%)	94	14	88	5
PPV (%)	79	22	81	15
snRNA			
Count	87		113	
Recall (%)	28	1	57	1
PPV (%)	67	7	67	9
transposon			
Count	187		361	
Recall (%)	77	5	80	24
PPV (%)	74	15	77	28
Overall			
Count	986		1190	
Accuracy (%)	81	33	79	33

Count is the number of loci present in that ncRNA class. Baseline performance is the performance obtained by randomly permuting the labels 100 times while keeping the class sizes the same.

For both high-depth data sets, the overall accuracy is ∼80%, which is a significant improvement over the baseline of 33%. The best performing classes are miRNA, C/D box snoRNA and transposon-derived RNAs. The performance of these three classes is also consistent between the two tissue types. In contrast, the lincRNA, scRNA and snRNA classes performed more poorly. The lower performance of these classes can possibly be attributed to their smaller representation among loci, as there were fewer smRNA loci present from these regions for both tissue types. Another potential reason for the lower performance is that these classes are less cohesive than the other classes. lincRNAs generally do not share any structural properties and are known to have diverse functional roles (25). The scRNAs are an umbrella group for two distinct types of RNAs: human Y RNAs and the BC200 scRNA (26), which have different secondary structures and likely different functions in the cell. Finally, the snRNA class is a highly incoherent group owing to the structural diversity among its members. For example, although the U1 and U2 RNAs are both small, localized to the nucleus, and involved in pre-mRNA splicing, they perform different functions and have different secondary structures (22). Therefore, it is reasonable to expect more diversity in the properties of smRNAs being produced by cleavage of snRNAs as opposed to the three better performing RNA classes.

### Features that can discriminate between classes of smRNAs

**Figure 6.**
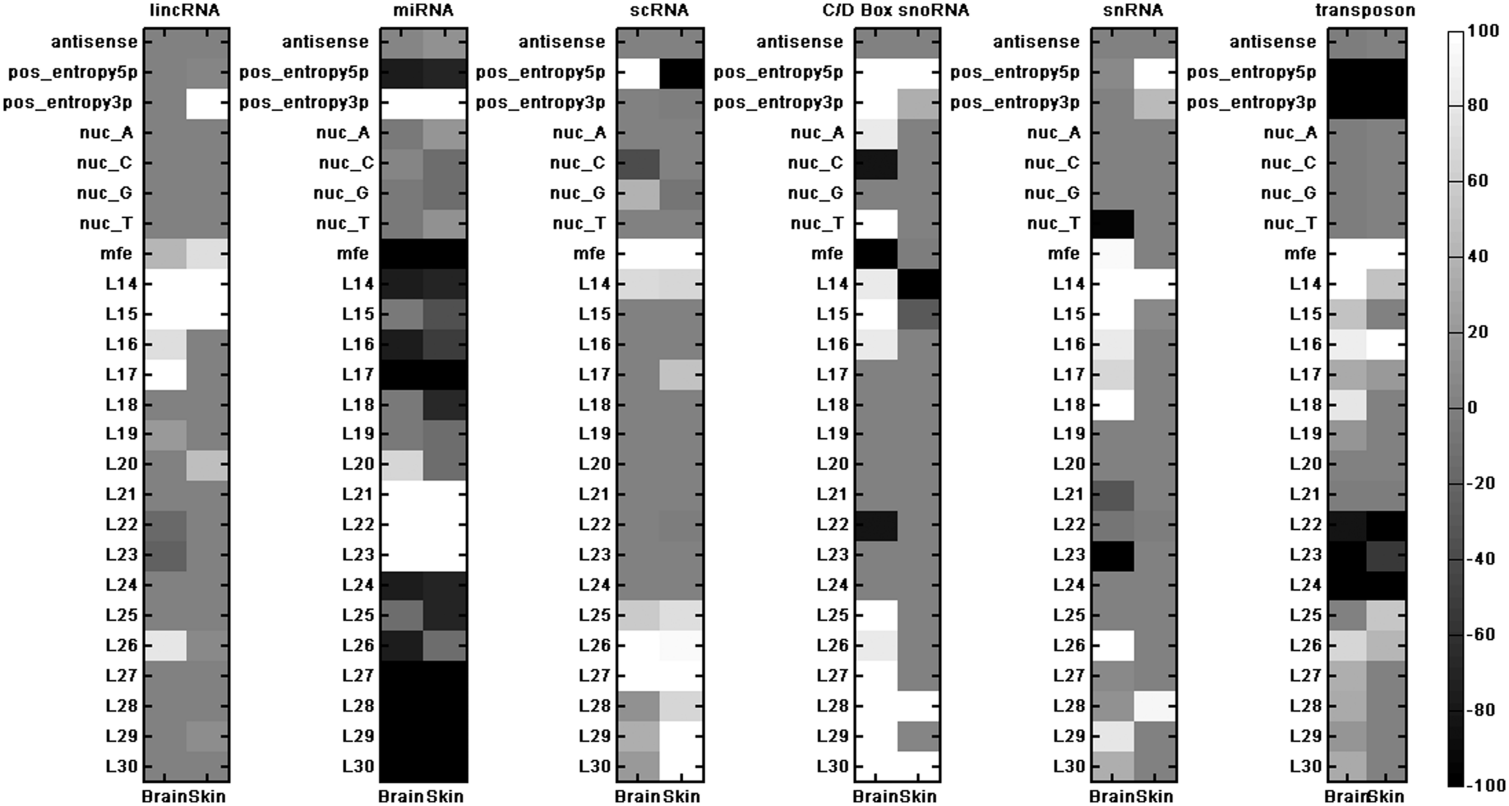
Selected features in each of the two data sets (as specified) for the six-class classifier: antisense expression (antisense), 5′ and 3′ smRNA positional entropy (pos_entropy5p and pos_entropy3p), nucleotide preference (nuc_A, nucC, nuc_G and nuc_T), MFE value and the smRNA length features from 14 to 30 nt (L14–L30). The sign of the value indicates whether the feature was larger (positive) or smaller (negative) within that class, on average, than the other classes (by difference of means).

Although we were interested in comparing the reproducibility of the smRNA features for various ncRNA classes, an important biological question to ask is which features are specific to which ncRNA classes. To determine this, we counted the number of times a feature is selected out of the 1000 RF models (Figure 6). To provide potentially biologically informative insights, we also marked features as being lower- or higher-valued in one class than in the others. We found that smRNAs from C/D box snoRNAs often have a higher positional entropy at their 5′ end and are short (<16 nt) or long (>25 nt). Interestingly, the length bias for these smRNAs is more marked in the brain data than in the skin data, but the entropy bias is consistent between tissues. The snRNAs do not have many discriminative features in the skin data set, but in the brain, they seem to preferentially produce shorter RNAs. Transposon-derived RNAs show low positional entropy—suggesting that their cleavage positions tend to be consistent. They also seem to be depleted of miRNA-length products (22–24 nt) while being enriched for shorter products (<19 nt) and having high MFE values for their secondary structure (Figure 6).

We found the class-specific features were largely consistent across the two tissues (human brain and skin) but vary widely for the ncRNA classes under study. For instance, lincRNAs show a propensity to produce shorter RNAs (14–17 nt), with slightly longer RNAs being produced in the skin data. Additionally, miRNAs were broadly distinguished by the production of fragments between 20 and 23 nt long, and this was consistent between the two tissue types. They also display a strong bias for low 5′ positional entropy and high 3′ entropy (Figure 6). This mirrors what is already known about lower variability of miRNA cleavage at the 5′ end and higher variability at the 3′ end (27).

The scRNA-derived smRNAs demonstrated a broad peak of discrimination at 27 nt for both tissue types, with skin RNAs showing longer lengths. It has previously been shown that Y RNA (a type of scRNA) fragments do produce miRNA-like smRNAs, but their potential function is still unclear (28). The scRNA-derived RNAs are moderately consistent between the two tissue types, but consistently show a preference for longer products with high MFE values (Figure 6).

Similar to scRNAs, C/D box snoRNAs were found to produce longer fragments. In both tissues, the positional entropy at both ends of the resulting smRNAs tended to be high, indicating a great degree of variability in cleavage positions. The pattern for snRNAs was less clear because their processing was highly inconsistent between the tissue types, with the exception of the production of 14 nt fragments, which was seen in both the brain and skin data sets (Figure 6). This may be due to the heterogeneity in the properties (especially structural) of RNAs that are collectively referred to as snRNAs. In contrast, we found that the features distinguishing transposable element-derived smRNAs were almost entirely consistent between the two tissues, with the most discriminative features being high cleavage specificity, high MFE, smaller products and the absence of miRNA-sized products (Figure 6). Thus, determining the mechanism of transposon-derived smRNA processing and their functions will likely be an interesting future research direction.

To determine whether a subset of features was the most useful for overall classification, we selected the first five dimensions from the MDS analysis. This resulted in a drop in overall accuracy of 8% (data not shown). This suggests that although a small number of features capture most of the differences between the classes, many other features are still highly informative. More importantly, results obtained from the original features are more conducive to interpretation than a model that is only generated based on a projection of the original features.

### Validation of the classification models between data sets

To evaluate the robustness of our classification models, we performed validation using independent data sets. To do this, we trained RF models on the brain data and applied them to the skin data and vice versa. Overall, the models were found to work fairly well, showing an accuracy of ∼80% in both cases (Table 3). This suggests that patterns of smRNAs produced from ncRNAs are generally consistent and mostly non-tissue specific. However, we found that the degree of consistency varies among the classes of smRNAs. The miRNAs, C/D box snoRNAs and transposon-derived RNAs show the most consistent results both within and between tissue types. However, the lincRNA and snRNA classes display tissue-specific patterns of smRNA processing (Table 3). This is expected for lincRNAs, given their tissue-specific patterns of expression. Besides tissue specificity, one other potential reason why certain classes perform much better across tissue types may be the number of loci present within the tissues being used for analysis. As we are using a fixed minimum of 15 reads mapping to each locus, differences in overall expression between the tissue types will result in a different number of loci in each class (Supplementary Figure S4). Therefore, although the cross-tissue classifier performs well overall, it is limited by not only the number of loci in each class but also the consistency in these numbers across the tissue types being studied.

To further validate the robustness of the classifier when applied to different data sets, we tested additional publicly available smRNA-seq data sets for human liver and muscle (Table 4). We restricted the classes to those represented by at least 10 loci in all four data sets (miRNA, C/D box snoRNA and tRNA). For each pair of data sets, we trained the model on one and tested on the other. Overall, the accuracies (65–93%) suggest that the model can classify across tissue types fairly well, conditional on the training data set having high enough sequencing depth to fully characterize the lower-abundance smRNAs. For example, the liver data set has far fewer reads than the others and thus performed poorest (<70%) when used as the training data set. Despite this, the model was able to classify liver smRNAs fairly well (77–93%) when tested on the other tissue types. Overall, our results suggest that CoRAL is a comprehensive and robust method for classifying RNAs using smRNA-seq data sets.

**Table 4.** Accuracy results for training classifiers on one tissue type and testing on another using the three-class model (miRNA, C/D box snoRNA, tRNA)

		Test			
		Brain	Skin	Liver	Muscle
Train	Brain	91%^a^	87%	93%	91%
	Skin	81%	89%^a^	81%	90%
	Liver	71%	67%	93%^a^	92%
	Muscle	63%	67%	93%	100%^a^

^a^Training accuracy.

## CONCLUSIONS

Patterns of cleavage in human ncRNAs appear to be non-random and reflect specificity in the processes that produce smRNAs from the corresponding precursors. This is despite the fact that the classes of ncRNAs studied here are defined based on differing criteria (sequence homology, secondary structure homology, biological function, cellular localization and transcript length). Although it is unknown whether these fragments or the cleavage of the precursors have any biological functions, the non-random nature of the cleavage events hints at some role.

We also found that the classification features that distinguished each class of ncRNA are generally consistent across tissue types in humans, suggesting there are as yet unknown biological pathways regulating their biogenesis. We also demonstrated that some types of ncRNAs show more tissue specific properties (lincRNAs, scRNAs and snRNAs). However, the other three RNA classes (miRNAs, C/D box snoRNAs and transposon-derived RNAs) are highly reproducible and consistent across two of the tissue types (brain and skin) tested in our study.

As compared with previous work like DARIO, one of the significant contributions of CoRAL is the development of biologically interpretable features such as fragment length, cleavage specificity and antisense transcription, which are able to capture the essence of ncRNAs (i.e. how they are processed into smaller fragments). It seems likely that the features revealed by CoRAL can serve as a basis for further exploration and validation.

The ability of CoRAL to consistently annotate loci between tissue types suggests that it may be useful in annotating ncRNAs in other organisms and even more tissue types using only smRNA-seq data. Thus, it will be a powerful tool for the annotation of future non-coding transcriptomes in this era of genomic progress, which complements other currently available comparative genomics methodologies. Our approach may even outperform homology-based methods, given the lower homology owing to compensatory evolution in many classes of RNAs (29).

## SOFTWARE AVAILABILITY

The CoRAL source code, required genome annotation files, and prediction results are available at http://wanglab.pcbi.upenn.edu/coral.

## SUPPLEMENTARY DATA

Supplementary Data are available at NAR Online: Supplementary Figures 1–4.

## FUNDING

National Institute of General Medical Sciences [R01-GM099962 to all coauthors]; the National Human Genome Research Institute [T32-HG000046 to P.R.]; the National Institute on Aging [U24-AG041689 and U01-AG032984 to L.S.W.]; the Penn Alzheimer’s Disease Center [P30-AG10124 to B.D.G., L.S.W. and Y.Y.L.] and the National Science Foundation [MCB-1053846 to B.D.G.]; Brain samples were obtained from the Center for Neurodegenerative Disease Research. Funding for open access charge: [R01-GM099962].

*Conflict of interest statement.* None declared.

## Supplementary Material

Supplementary Data
