# Supplementary material for: CoRAL: predicting non-coding RNAs from small RNA-sequencing data
Source: Nucleic Acids Res. 2013 May 21;41(14):e137. doi: 10.1093/nar/gkt426 (PMC3737537; doi:10.1093/nar/gkt426)
Supplement: Supplementary Data [file supp_41_14_e137__index.html]

CoRAL: predicting non-coding RNAs from small RNA-sequencing data — CoRAL: predicting non-coding RNAs from small RNA-sequencing data — Supplementary Data 

# CoRAL: predicting non-coding RNAs from small RNA-sequencing data

## Supplementary Data

files

**Files in this Data Supplement:**

- Supplementary Data - doc file
- Supplementary Data - tiff file
- Supplementary Data - tiff file
- Supplementary Data - tiff file
- Supplementary Data - tiff file
