# Supplementary material for: CoRAL: predicting non-coding RNAs from small RNA-sequencing data
Source: Nucleic Acids Res. 2013 May 21;41(14):e137. doi: 10.1093/nar/gkt426 (PMC3737537; doi:10.1093/nar/gkt426)

**Supplementary Fig. 1: The effect of read counts on class sizes in the smRNA-seq datasets for two human tissue types: a) brain, and b) skin.**

1. ***brain***

***
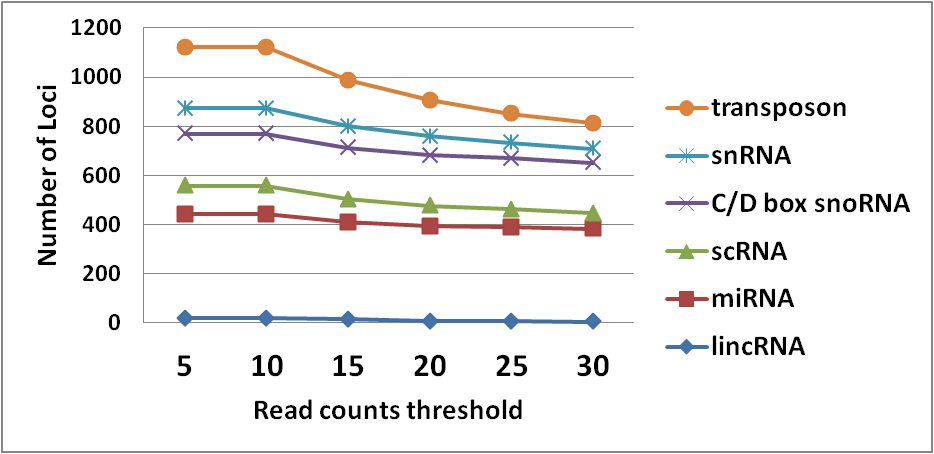
***

1. ***skin***

***
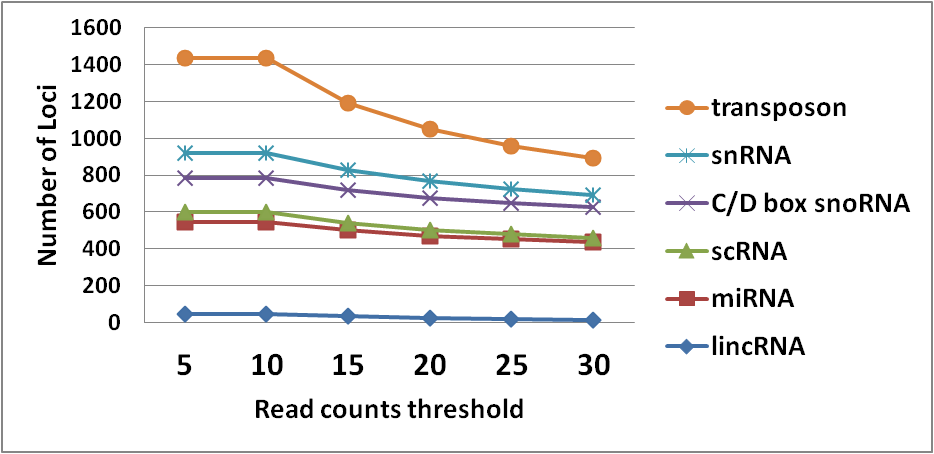
***

**Supplementary Fig. 2: Feature spectrum plots for three of the ncRNA classes (as specified in the figure), in the a – c) brain data, and d – f) the skin data. Each box corresponds to one length feature and each gray line represents one locus. The red dots are outside of the 99^th^ percentile of each distribution.**

**lincRNA**

**a) d)**

**
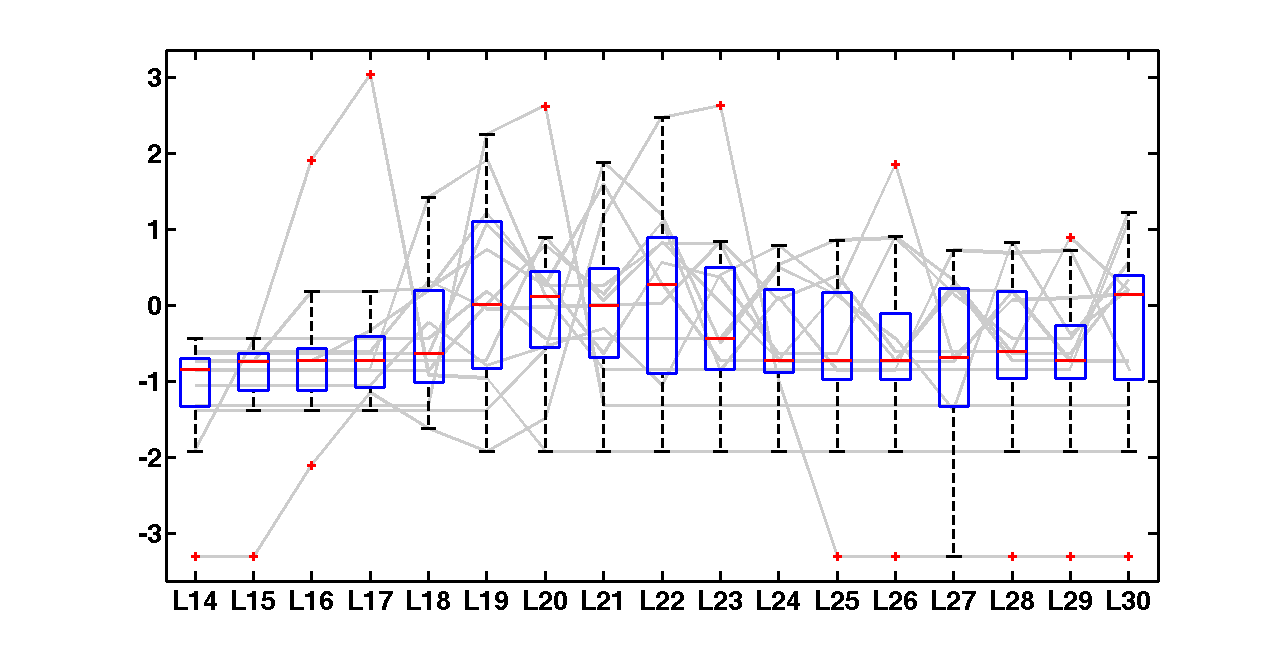

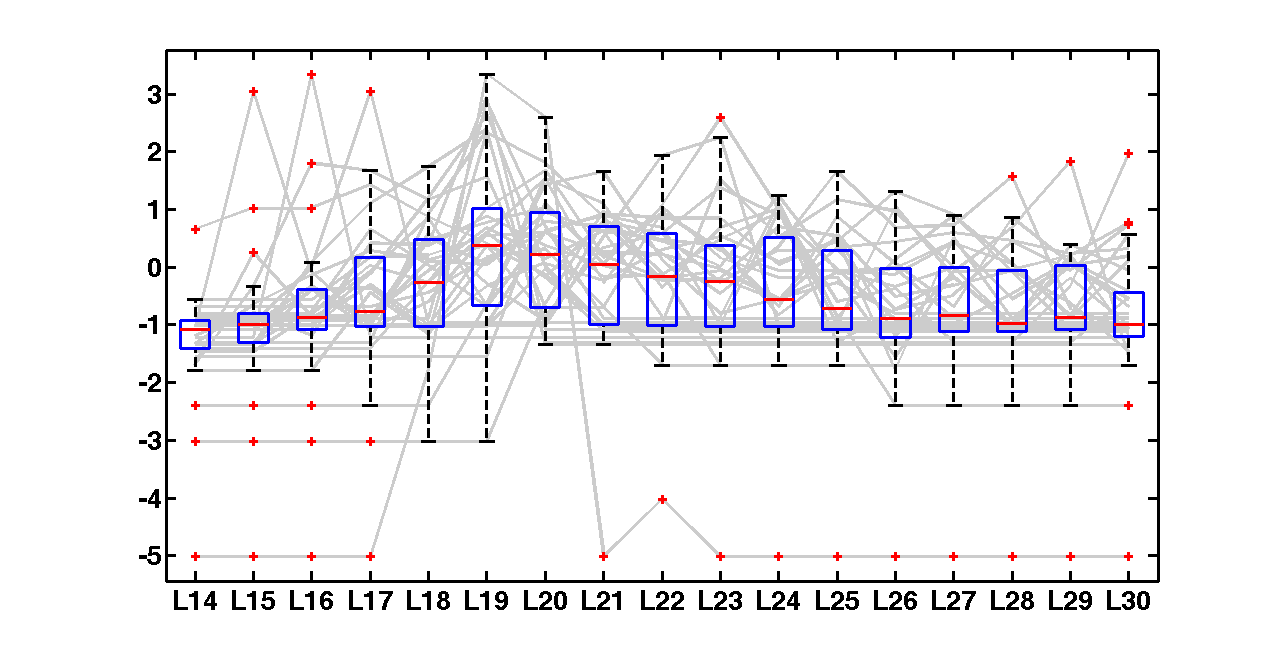
**

**scRNA**

**b) e)**

**
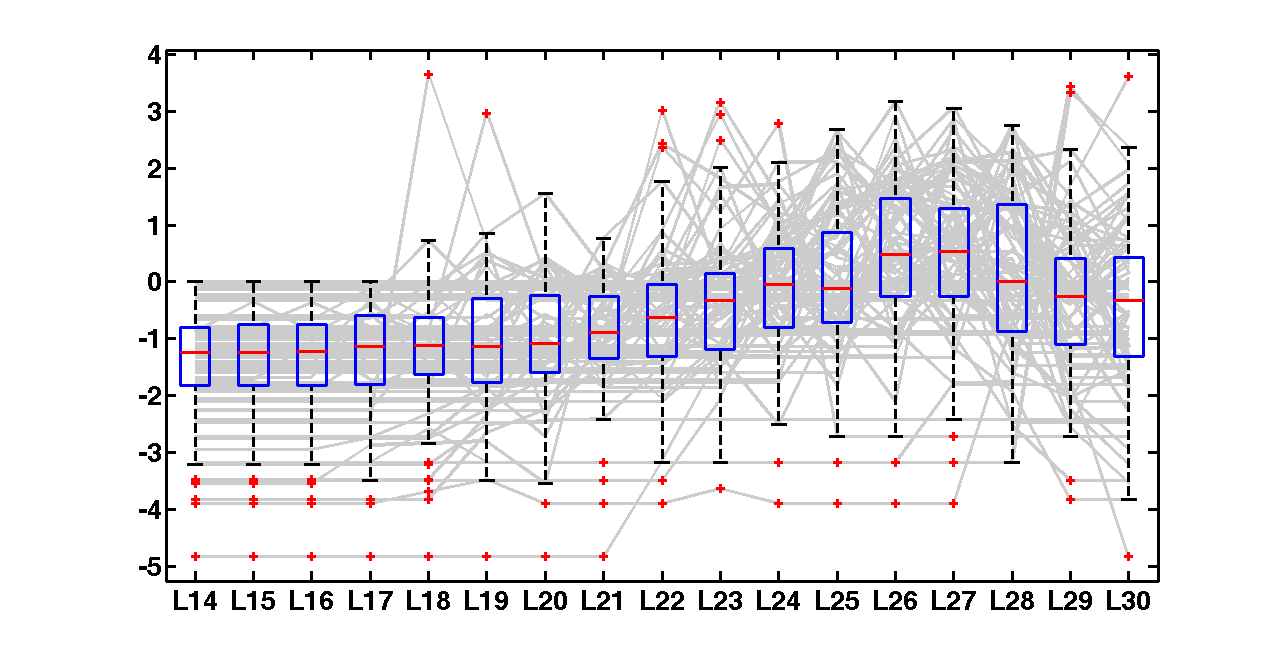

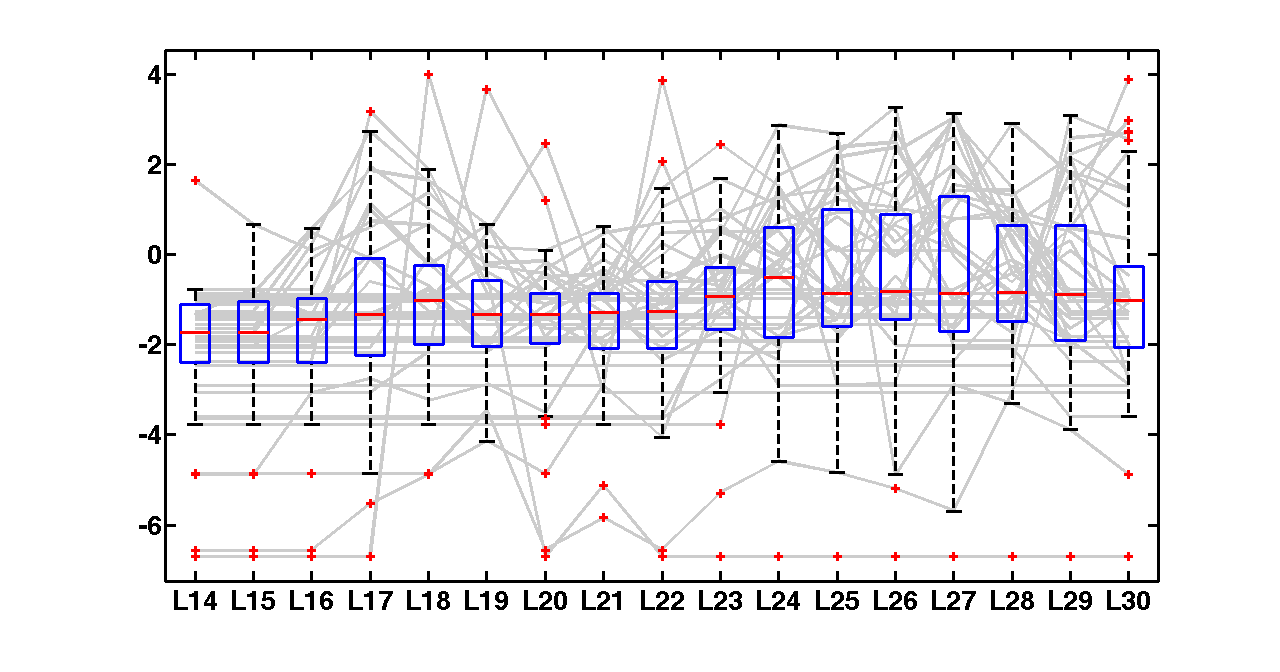
**

**snRNA**

**c) f)**

**
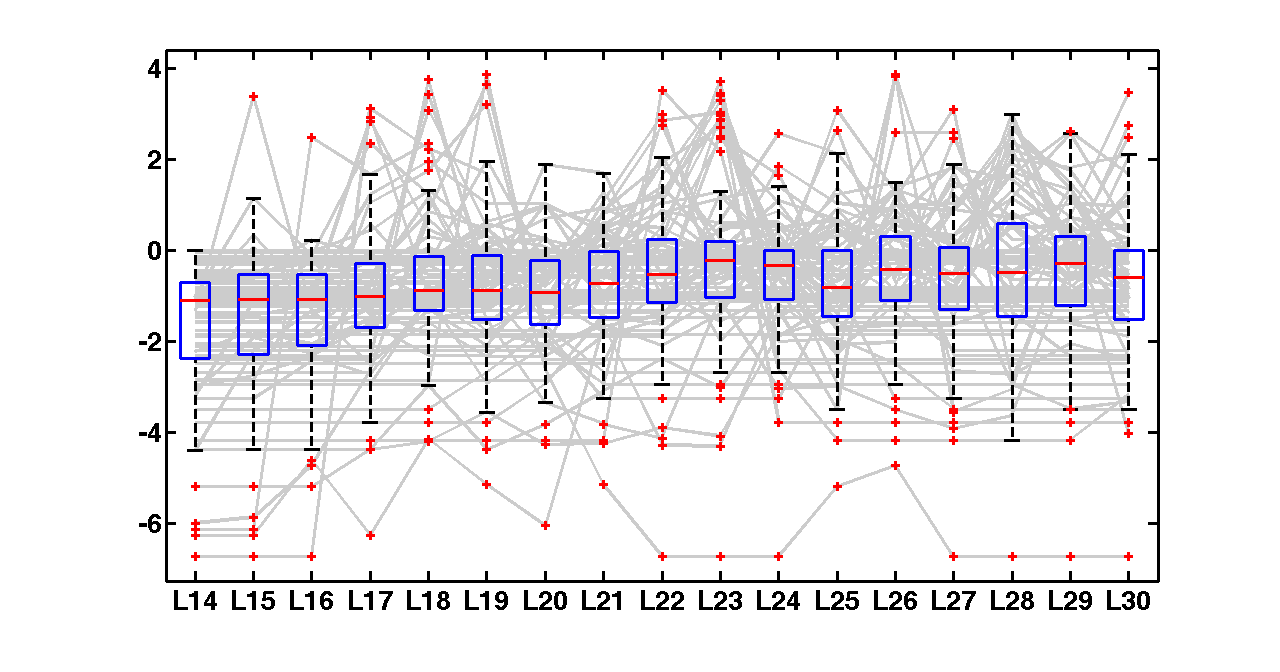

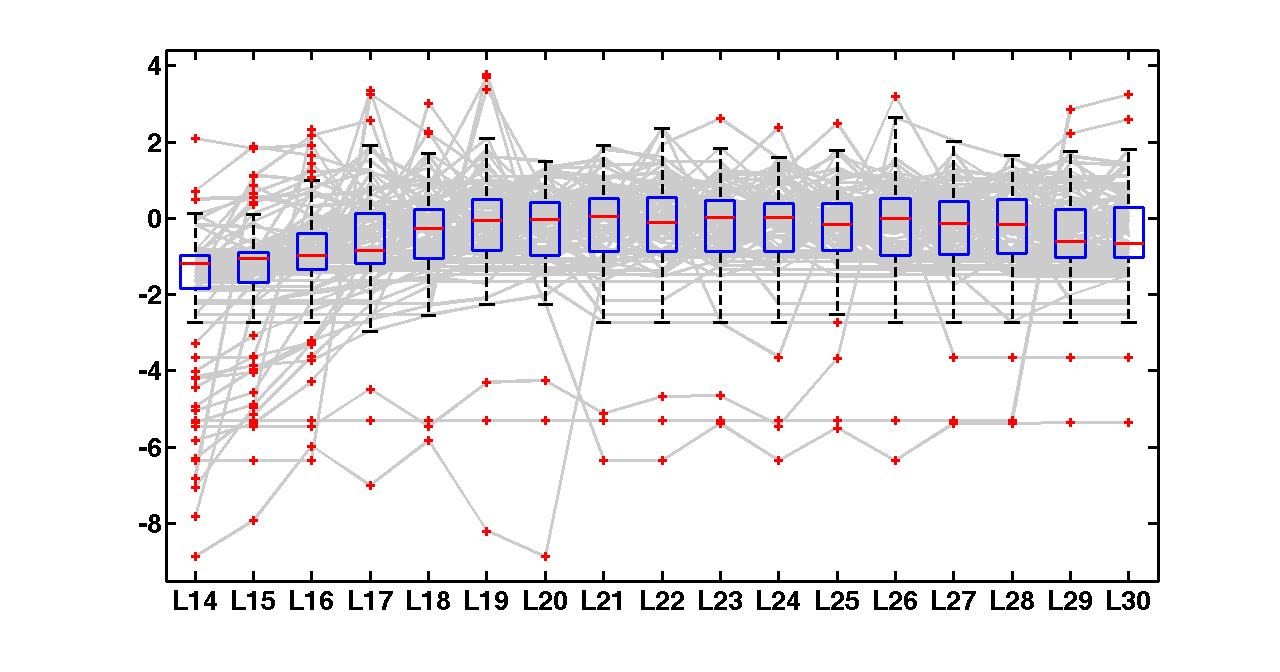
**

**Supplementary Fig. 3: Correlation heatmap of the features in the brain data.**

**
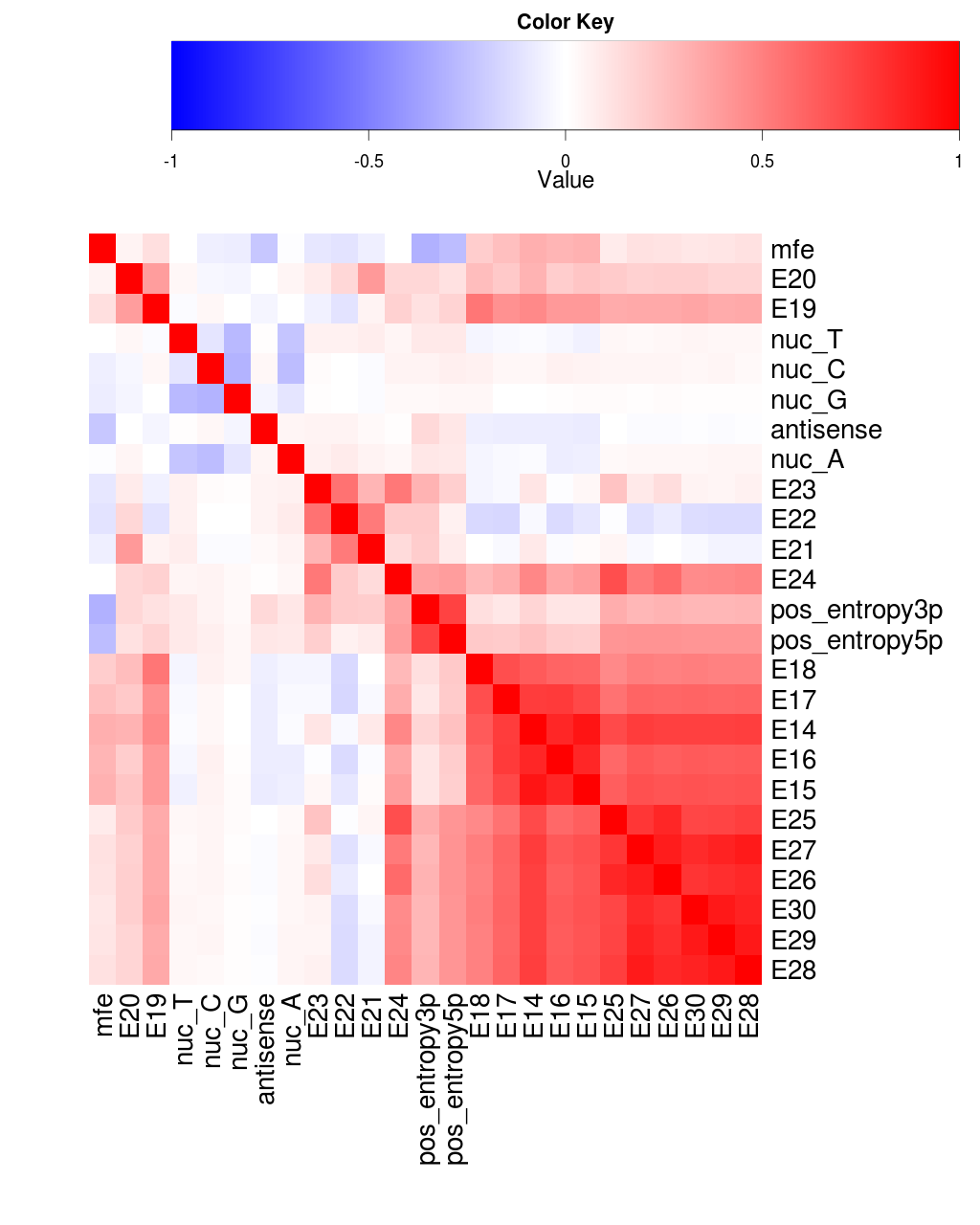
**

**Supplementary Fig. 4: Venn diagrams for the overlap of each type of ncRNA between the brain and skin datasets.**

1. lincRNA b) miRNA c) scRNA


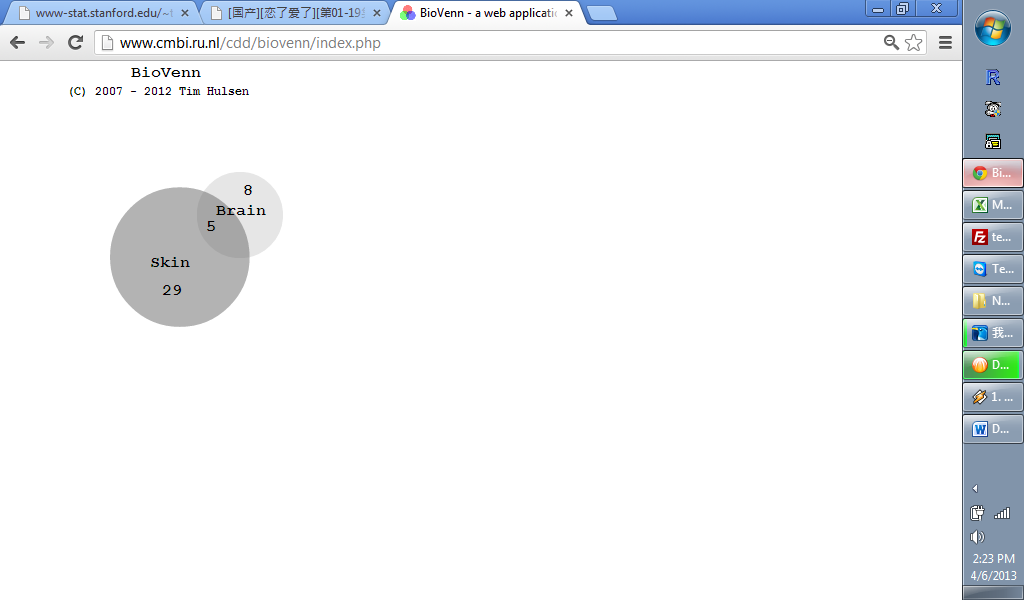

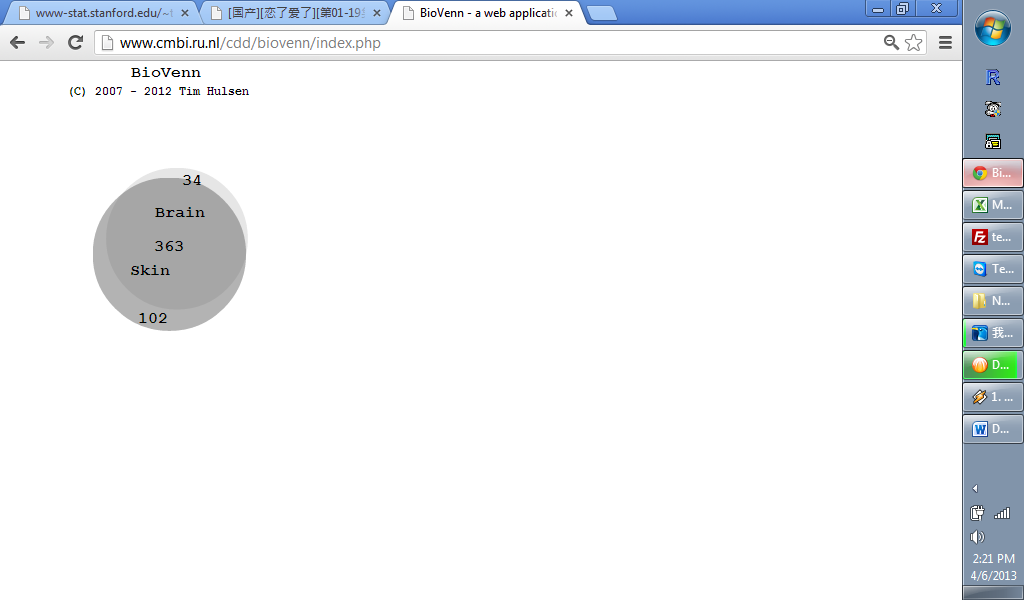

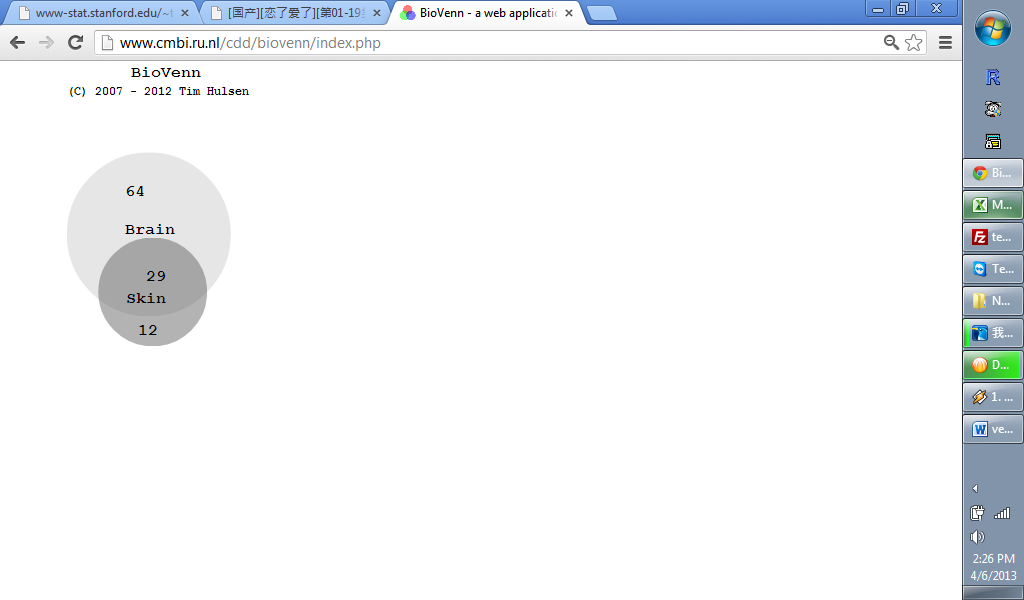


1. C/D box snoRNA e) snRNA f) transposon


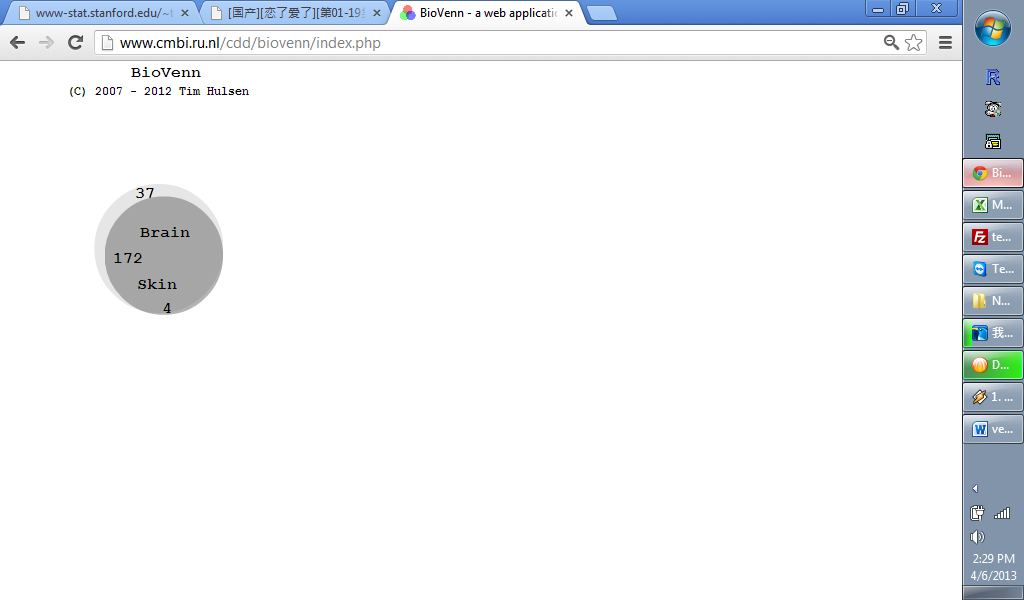

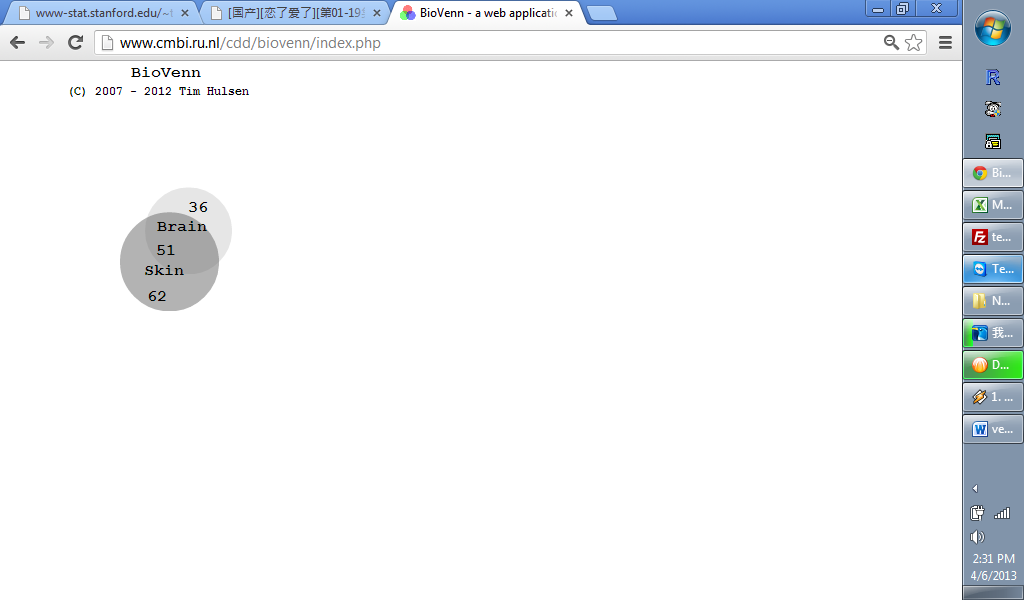

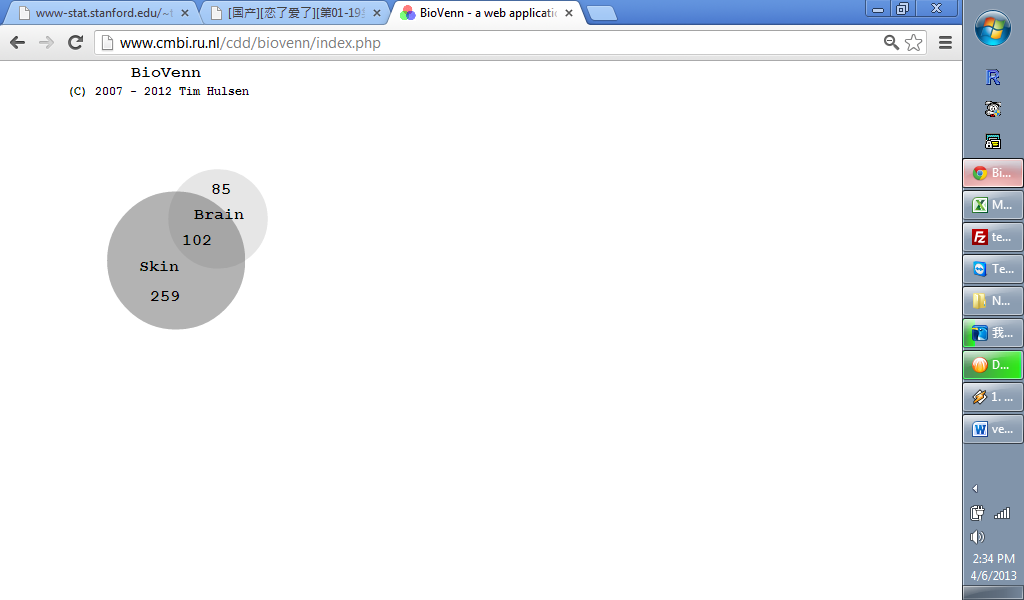

Supplement: Supplementary Data [file supp_gkt426_nar-00046-met-n-2013-File002.doc]
