# Supplementary figures and images for: CoRAL: predicting non-coding RNAs from small RNA-sequencing data
Source: Nucleic Acids Res. 2013 May 21;41(14):e137. doi: 10.1093/nar/gkt426 (PMC3737537; doi:10.1093/nar/gkt426)

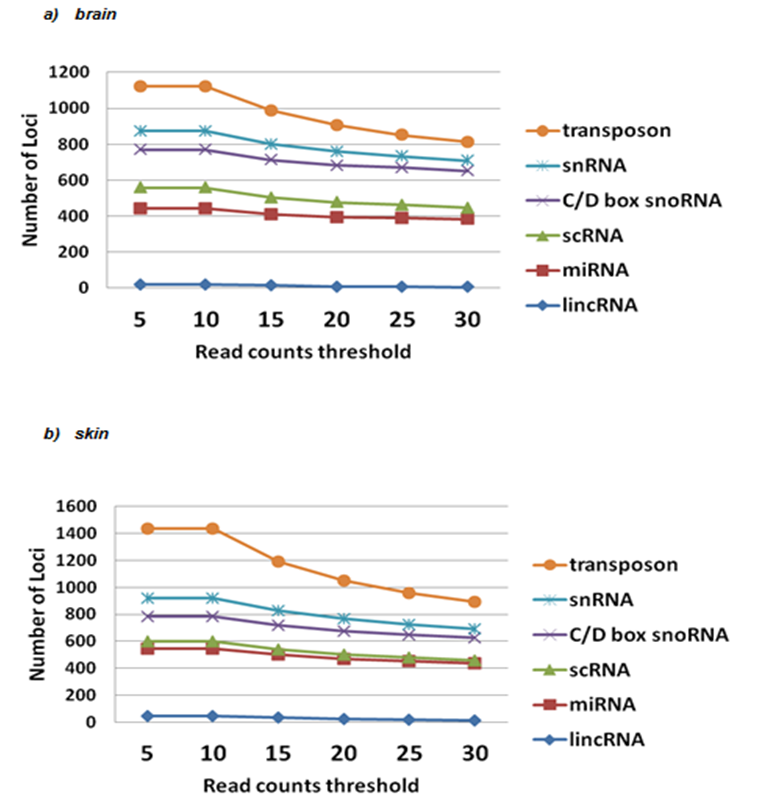

Supplement: Supplementary Data [file supp_gkt426_nar-00046-met-n-2013-File009.tif]

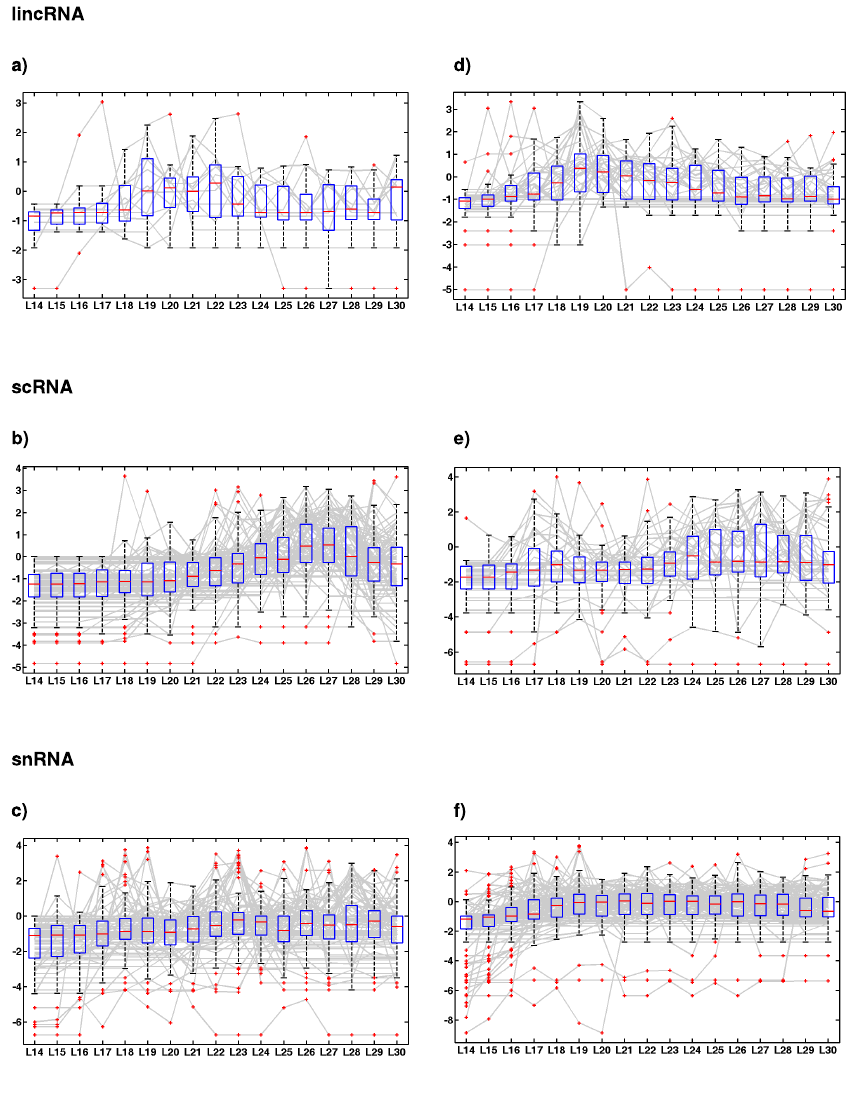

Supplement: Supplementary Data [file supp_gkt426_nar-00046-met-n-2013-File010.tif]

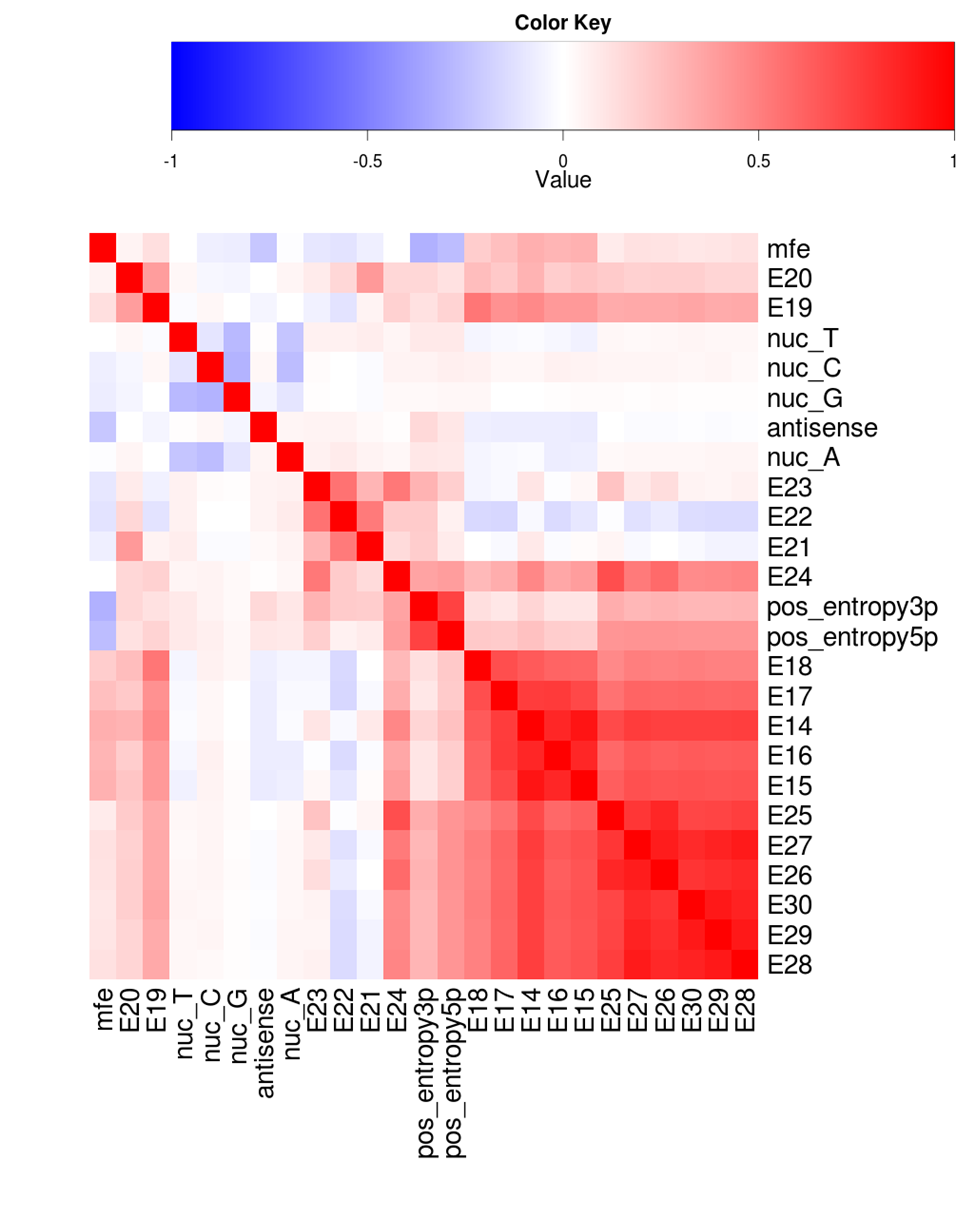

Supplement: Supplementary Data [file supp_gkt426_nar-00046-met-n-2013-File011.tif]

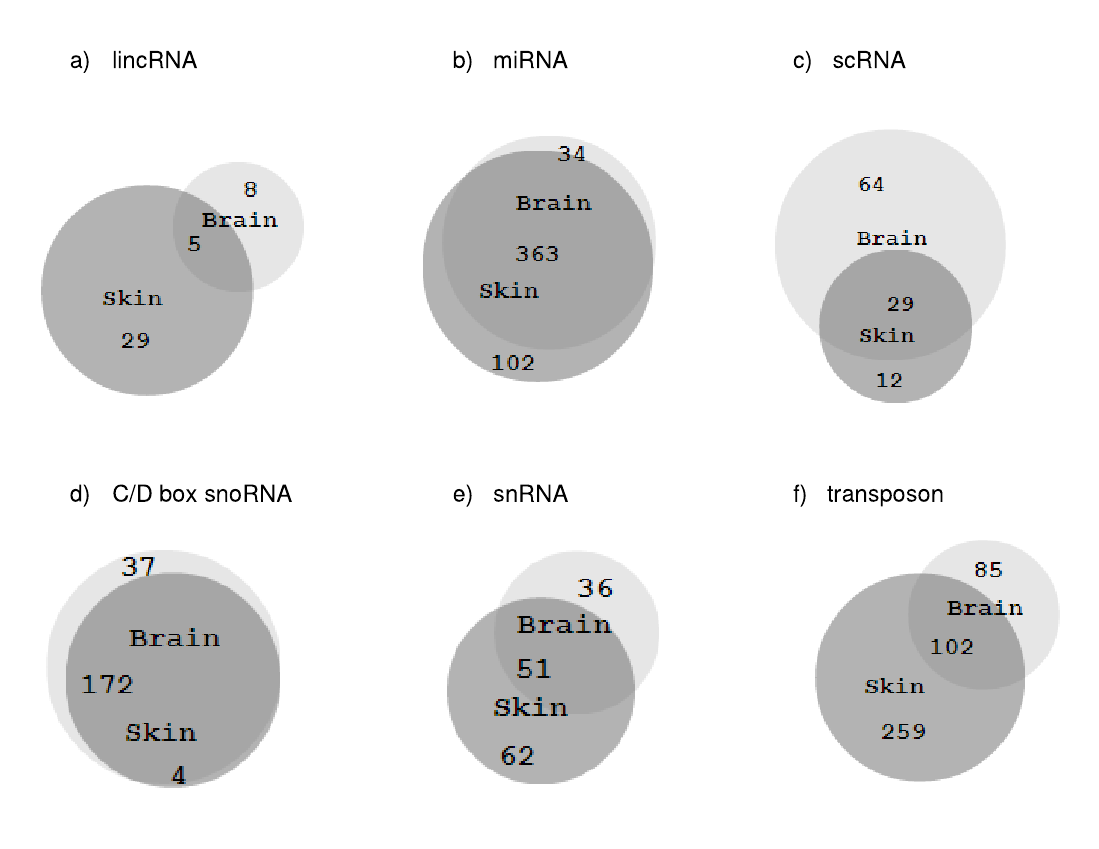

Supplement: Supplementary Data [file supp_gkt426_nar-00046-met-n-2013-File012.tif]
